# Renal Extramedullary Hematopoiesis in Mast Cell Leukemia with Bone Marrow Fibrosis

**DOI:** 10.1155/2024/3502887

**Published:** 2024-01-03

**Authors:** Damian T. Rieke, Laura K. Schmalbrock, Jana Ihlow, Karsten Kleo, Ann-Christin von Brünneck, Florian Nolte, Ulrich Keller, Sebastian Ochsenreither

**Affiliations:** ^1^Department of Hematology, Oncology and Cancer Immunology, Campus Benjamin Franklin, Charité ‐ Universitätsmedizin Berlin, Hindenburgdamm 30, Berlin 12203, Germany; ^2^Berlin Institute of Health (BIH) at Charité, Universitätsmedizin Berlin, Anna-Louisa-Karsch-Straße 2, Berlin 10178, Germany; ^3^German Cancer Consortium (DKTK) Partner Site Berlin, German Cancer Research Center (DKFZ), Heidelberg, Germany; ^4^Institute of Pathology, Charité ‐ Universitätsmedizin Berlin, Charitéplatz 1, Berlin 10117, Germany; ^5^Praxis Onkologie Seestrasse, Seestrasse 64, Berlin 13347, Germany

## Abstract

Systemic mastocytosis is defined by the clonal proliferation of abnormal mast cells. The clinical course can range from indolent forms with normal life expectancy to advanced mast cell leukemia with dismal prognosis. An association with other diseases, including myeloproliferative neoplasia, has been described. We present a case of a 75-year patient with a history of cutaneous mastocytosis who was diagnosed with mast cell leukemia more than 9 years ago and did not receive treatment. The patient presented to our clinic with acute kidney failure because of renal extramedullary hematopoiesis. Bone marrow histopathology revealed extensive fibrosis and 50% infiltration by mast cells with a c*-KIT* D816V mutation. No mutations supporting primary myelofibrosis were identified. Treatment with midostaurin was started, and the patient was discharged after improvement of renal function. Here, we discuss diagnostic challenges between different forms of mast cell leukemia and overlaps with other hematological malignancies.

## 1. Introduction

Systemic mastocytosis (SM) results from the clonal proliferation of abnormal mast cells [1]. Published international consensus (ICC) and world health organization (WHO) classifications define SM by multifocal, dense infiltrates of tryptase and/or CD117-positive mast cells in bone marrow (BM) and/or other extracutaneous organs together with minor criteria, such as a point mutation at codon 816 of *c-KIT*. Isolated BM mastocytosis is described as a variant in the ICC, whereas it has been refined as a separate subcategory in the current WHO classification [2, 3]. SM is frequently associated with other hematological neoplasms, thus defining the separate subentity of systemic mastocytosis with an associated hematological neoplasm (SM-AHN) in the WHO classification, which falls into the ICC category of advanced mastocytosis with associated myeloid neoplasms (SM-AMN) [1–3]. In SM, the degree of severity ranges from indolent SM to mast cell leukemia (MCL) with a dismal prognosis [4]. MCL is defined by the presence of at least 20% immature atypical mast cells in the BM smear and/or diffuse infiltration by atypical mast cells in the BM biopsy, in addition to SM criteria [1] or diffuse infiltration of atypical mast cells in the BM biopsy [2, 3]. MCL can be further subdivided into leukemic and aleukemic subtypes as well as in acute and chronic MCL. The majority of MCL patients show acute MCL with dismal prognosis and organ damage defined by C-findings. In contrast, chronic MCL is defined by the absence of C-findings [5]. Progression of these patients to acute MCL with BM infiltration and extramedullary infiltration is very common [6]. A clear morphology-based definition of chronic MCL is still lacking. In the light of this recurring challenge, we report and discuss the case of a 75-year-old woman with extramedullary hematopoiesis following BM fibrosis in MCL.

## 2. Case Presentation

A 75-year-old female patient presented to the emergency department with dyspnea and weakness progressing over several weeks. The patient lived alone and had received a diagnosis of cutaneous mastocytosis more than 20 years and of MCL 9 years ago (30% mast cell BM infiltration). She reported not having gone to follow-up visits because she was unaware of her diagnosis. Physical examination revealed a fully oriented but pale patient with a reduced performance status (ECOG 2-3) and peripheral pitting edema as well as brownish discolorations of the left upper thigh. Laboratory tests revealed severe anemia (Hb 2.4 g/dl, reference 11.8–15.8), thrombocytopenia (22/nl, reference 150–370), and leukocytosis (62/nl, reference 3.9–10.5), as well as renal failure (creatinine 4.55 mg/dl, reference 0.5–0.9; urea 315 mg/dl, reference 17–48) with proteinuria, leukocyturia, and hematuria. C-reactive protein was increased (59.4 mg/l, reference <5).

**Figure 1:**
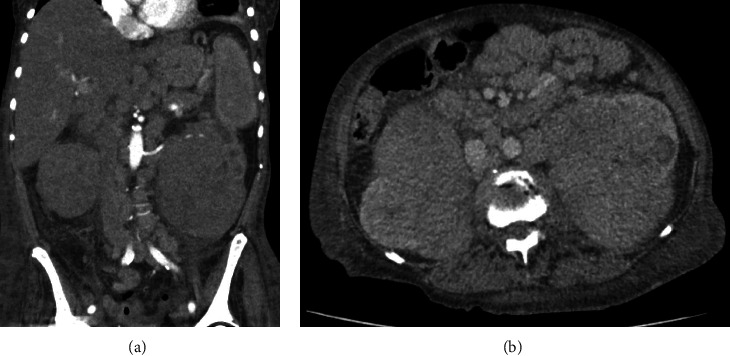
CT scan showing renal/pararenal masses in the patient. (a) Abdominal coronal plane, showing masses with near-total compression/infiltration of the kidneys. Remaining renal structures of the left kidney are visible in this section. (b) Transverse plane of the same patient.

Emergency esophagogastroduodenoscopy showed diffuse superficial erosive gastritis and bulbitis without signs of acute bleeding. Sonography and a CT scan showed bilateral pararenal/renal masses (Figure 1), whereby the renal pelvis could not be adequately evaluated.

**Figure 2:**
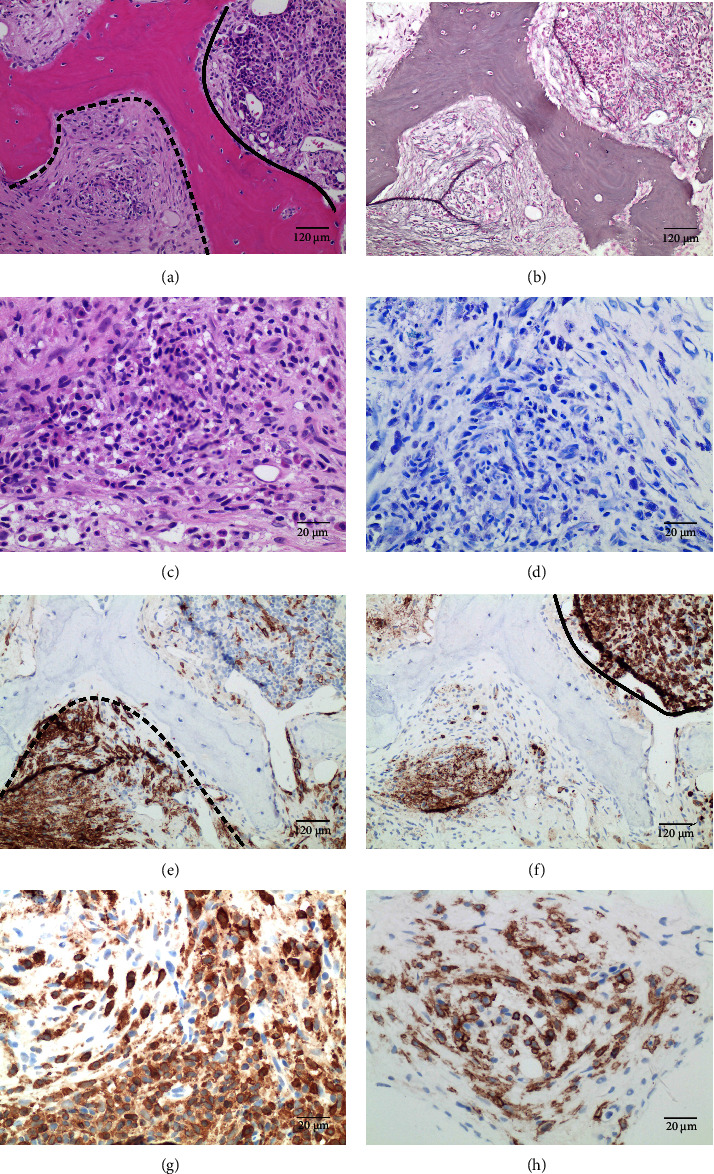
Bone marrow biopsy reveals mastocytosis. (a) H&E stain shows a patchy distribution of areas with atypical mast cell infiltrates, concentric fibrosis (dashed line), and granulocyte-rich areas (bold line). Trabeculae shows signs of beginning osteosclerosis with focal spikes and paratrabecular apposition of new bone (osteosclerosis grade 1). (b) Gomori stain damasks a diffuse and dense increase in reticulin with extensive intersections, occasionally with focal bundles of thick fibres, associated to focal myelofibrosis (MF-2), particularly in the mast cell-rich area. (c) H&E stain shows infiltrates of atypical spindle-shaped mast cells without metachromatic granules and without blast-like appearance. (d) Giemsa stain exposes atypical mast cells, some of which still contain metachromatic granules. (e) Immunohistochemical staining for CD117 highlights atypical mast cell infiltrates (encircled by the dashed line). (f) Immunohistochemical staining for MPO highlights patchy granulocyte-rich area (encircled by a bold line). (g) Expression of mast cell tryptase is present within the atypical mast cell aggregates. (h) CD25 expression emphasizes the atypical immunophenotype of the mast cells.

The patient was admitted to the intensive care unit. Erythrocyte and platelet transfusions, high-dose proton-pump inhibitor treatment, and empirical antibiotic therapy with piperacillin/tazobactam were started for suspected complicated urinary tract infection. Furthermore, hemodialysis was initiated over three days. Following microbiological evidence of E. coli infection in urine and blood samples, antibiotic treatment was changed to cefotaxim. Helicobacter pylori antigen was negative in repeated stool samples. Bilateral double-J catheters were placed after which purulent urine drained. Retrograde intraoperative imaging revealed kinking of the ureter and displacement of the renal pelvis caused by the large pararenal lesion. Differential blood count showed an increase in neutrophils, lymphocytes, and monocytes. A peripheral blood smear revealed an absence of blasts and mast cells. Serum tryptase was elevated (64.5 *μ*g/l, reference <11.4). Due to punctio sicca, cytology could not be assessed. BM biopsy revealed 50% infiltration by aggregates of atypical, mostly spindle-shaped nonblast-like mast cells (CD25/CD117+) without metachromatic granules. Furthermore, there was concomitant patchy BM fibrosis grade MF-2 according to WHO (Figure 2). Using a customized MDS-panel for next-generation sequencing on the FFPE-BM biopsy, we detected a *c-KIT* p.D816V mutation with an allele frequency (AF) of 37%. Moreover, *TET2* p.R1516^*∗*^ (AF 78%), *ASXL1* p.E1132K (AF 5.6%), and *CSF3R* c.2041-8C > T (AF 48%) variants were detected.

**Figure 3:**
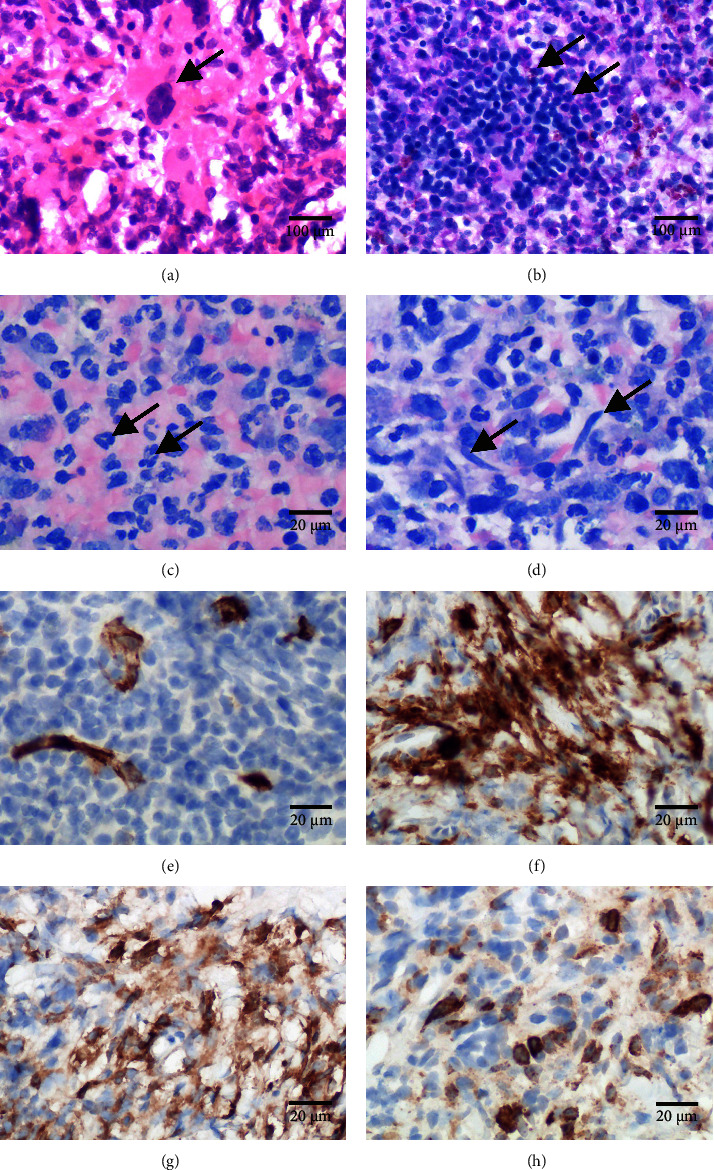
Biopsy of the renal mass reveals extramedullary hematopoiesis and only few mast cell aggregates. (a, b) H&E stain shows extramedullary hematopoiesis. The single arrow in panel (a) illustrates megakaryopoiesis and double arrows in panel (b) indicate a mature group of erythroblasts. (c, d) Giemsa stain shows extramedullary granulopoiesis. Arrows in panel (c) depict segmented mature granulocytes, and arrows in panel (d) highlight intermixed atypical spindle-shaped mast cells. (e) Immunohistochemical staining for CD34 highlights vascular endothelium and shows absence of blasts. (f) Immunohistochemical staining for CD117 reveals mast cell hotspots. (g) Mast cell tryptase supports the presence of mast cells. (h) Only few mast cells show expression of CD25.

We performed a sonography-guided biopsy of the renal/pararenal mass in which extramedullary hematopoiesis with less than 5% interspersed spindle-shaped mast cells became apparent (Figure 3).

We made a diagnosis of aleukemic MCL with extramedullary hematopoiesis leading to renal compression and acute renal failure. The patient`s medical history comprised cutaneous mastocytosis more than 20 years ago and a diagnosis of chronic MCL with proof of c-*KIT* p.D816V and *TET2* p.R1516^*∗*^ mutation 9 years ago, in line with the current diagnosis. Treatment with midostaurin (50 mg bid) was started [7]. The patient could eventually be discharged with an improved performance status.

**Figure 4:**
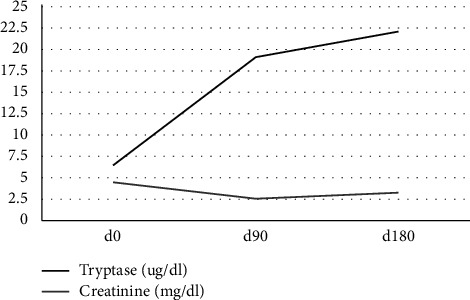
Timeline of serum creatinine (reference range 0.5–0.9) and tryptase (reference range <1.14) levels between time of initial presentation (d0) and time of death (approx. d180).

Unfortunately, the patient was admitted to the hospital again 6 months later with acute kidney failure and urosepsis and succumbed to this complication. Serum creatinine and tryptase levels between emergency unit presentation and time of death are provided in Figure 4. Autopsy confirmed MCL with lymphadenopathy and hepatomegaly with mast cell infiltrations as well as extramedullary hematopoiesis.

## 3. Discussion

MCL is a rare form of advanced SM, defined by the presence of at least 20% atypical mast cells in the BM smear in addition to SM criteria [1]. As per ICC 2022, diagnosis can be based on the presence of diffuse atypical mast cell infiltrates of at least 25% in the BM biopsy alone in the case of dry BM aspiration, which justified the diagnosis in our patient [2]. Following the proposed categories of MCL [1, 5, 8], this patient presented with an aleukemic variant, and despite harboring C-findings (cytopenia, organomegaly) showed a chronic course given the primary diagnosis 9 years prior to presentation. A cutaneous mastocytosis had been reported more than 20 years ago. This is also in line with the clinically suspected cutaneous involvement of the upper left thigh, which was not biopsied for a lack of therapeutic consequences. Comparably lower serum tryptase and absence of signs of mast cell activation were also notable in this patient [9]. Furthermore, despite diffuse gastritis, no gastroduodenal ulcer were found. Yet, the expression of CD25 and the typical c*-KIT* mutation all clearly indicate atypical mast cells in this patient. This patient's disease course is exceptionally long, spanning at least 9 years without having received any treatment. This far exceeds the median survival times of 5 months to 1.6 years in patients with MCL [5, 9], especially in consideration of previous reports associating *TET2*-mutations with more aggressive SM [10]. In addition, this patient is older than most previously reported patients with this disease [5, 9]. Together with the more mature morphology detected in the BM biopsy, our findings suggest a disease biology less aggressive than in most MCL. In addition, coexisting myelofibrosis is not typically described in MCL.

SM with associated hematological neoplasm (SM-AHN) has been described as a distinct subtype of SM, including patients with chronic idiopathic myelofibrosis [1, 11]. In these patients, an accompanying *JAK2*-mutation was also identified in the mast cells, supporting the diagnosis of underlying myeloproliferative disease (MPN). In our patient, neither *JAK2*, *MPL*, *CALR* mutation nor *BCR-ABL1* fusion was identified. C*SF3R* mutations have rarely been described in myeloid malignancies, including MPN [12], yet the here identified splice site variant is of unclear significance (VarSeak class 2, likely no splicing effect). Prominent leukocytosis and remaining granulopoiesis in the BM additionally suggest a myeloproliferative aspect, similar to a previously described type 2 infiltration pattern, which was albeit associated with poor prognosis [13]. Yet, secondary reactive myelofibrosis due to mast cell-associated fibrosis might be a more likely underlying mechanism and has been described accordingly [14].

Thus, the long clinical course and clinical and molecular findings suggest a diagnosis of SM-AHN in contrast to pure MCL, highlighting the difficulty of exact diagnosis in this rare disease. Identified molecular alterations and monocytosis might favor coexisting chronic myelomonocytic leukemia (CMML) as associated hematological neoplasm.

The extramedullary hematopoiesis therefore follows a long and chronic disease course with progressive BM fibrosis. Renal/pararenal extramedullary hematopoiesis is a rare finding, which is in most cases accompanied by renal failure [15] and usually associated with hematologic disease other than SM, most commonly MPNs [15–17]. Extramedullary hematopoiesis has been described in about 20% of patients with SM [18], even though renal extramedullary hematopoiesis has, to the best of our knowledge, not been described in MCL.

These findings further indicate a clinical overlap of SM/MCL and myeloproliferative features as seen in our patient with C-findings rather caused bycomplications of chronic disease. These considerations give rise to the question whether the absence of C-findings is sufficient for a diagnosis of chronic MCL [3, 19]. Our data strongly highlight the need to correlate morphology and molecular results to the clinical course of the disease in larger cohorts to refine the diagnostic distinction between chronic MCL, acute MCL, and overlap cases. The presence of associated hematological neoplasms in SM-AHN—as potentially present in the here presented patient—additionally requires careful consideration. This might hold important consequences for patient treatment and follow-up in this otherwise aggressive disease.

## Data Availability

The data supporting the conclusions of this study are provided in the manuscript text and figures.
